# Evaluation of two strategies for the interpretation of tumour markers in pleural effusions

**DOI:** 10.1186/s12931-017-0582-1

**Published:** 2017-05-25

**Authors:** Jaume Trapé, Francesc Sant, Josefina Franquesa, Jesús Montesinos, Anna Arnau, Maria Sala, Oscar Bernadich, Esperanza Martín, Damià Perich, Concha Pérez, Joan Lopez, Sandra Ros, Enrique Esteve, Rafael Pérez, Jordi Aligué, Gabriel Gurt, Silvia Catot, Montserrat Domenech, Joan Bosch, Josep Miquel Badal, Mariona Bonet, Rafael Molina, Josep Ordeig

**Affiliations:** 1 Department of Laboratory Medicine, Althaia Xarxa Assistencial Universitària de Manresa, Dr Joan Soler 1-3, 08243 Manresa, Catalonia Spain; 2 Department of Pathology, Althaia Xarxa Assistencial Universitària de Manresa, Manresa, Catalonia Spain; 3 Department of Oncology, Althaia Xarxa Assistencial Universitària de Manresa, Manresa, Catalonia Spain; 4 Clinical Research Unit, Althaia Xarxa Assistencial Universitària de Manresa, Manresa, Catalonia Spain; 5 Department of Pulmonary Diseases, Althaia Xarxa Assistencial Universitària de Manresa, Manresa, Catalonia Spain; 6 Department Internal Medicine, Althaia Xarxa Assistencial Universitària de Manresa, Manresa, Catalonia Spain; 7 Polytechnic School of University of Vic, Universitat Central de Catalunya, Vic, Catalonia Spain; 8 Department of Medicine, Universitat Internacional de Catalunya, Sant Cugat, Catalonia Spain; 9 Laboratory of Biochemistry (Oncobiology Unit), Biomedical Diagnostic Center (CDB), Hospital Clinic, IDIBAPS, University of Barcelona, Catalonia, Spain

**Keywords:** Pleural effusion, Diagnostic, Cancer, Tumour markers

## Abstract

**Background:**

Pleural effusions present a diagnostic challenge. Approximately 20% are associated with cancer and some 50% require invasive procedures to perform diagnosis. Determination of tumour markers may help to identify patients with malignant effusions. Two strategies are used to obtain high specificity in the differential diagnosis of malignant pleural effusions: a) high cut-off, and b) fluid/serum (F/S) ratio and low cut-off. The aim of this study is to compare these two strategies and to establish whether the identification of possible false positives using benign biomarkers – ADA, CRP and % of polymorphonuclear cells – improves diagnostic accuracy.

**Methods:**

We studied 402 pleural effusions, 122 of them malignant. Benign biomarkers were determined in pleural fluid, and CEA, CA72-4, CA19-9 and CA15-3 in pleural fluid and serum.

**Results:**

Establishing a cut-off value for each TM for a specificity of 100%, a joint sensitivity of 66.5% was obtained. With the F/S strategy and low cut-off points, sensitivity was 77% and specificity 98.2%, Subclassifying cases with negative benign biomarkers, both strategies achieved a specificity of 100%; sensitivity was 69.9% for single determination and 80.6% for F/S ratio.

**Conclusions:**

The best interpretation of TM in the differential diagnosis of malignant pleural effusions is obtained using the F/S ratio in the group with negative benign biomarkers.

## Background

Pleural effusions present a diagnostic challenge. Between 15 and 30% are associated with cancer and between 40 and 60% require invasive procedures to perform diagnosis [1–3]. The clinical utility of tumour markers (TM) in the differential diagnosis of malignancy in pleural effusions is controversial. In the literature a wide range of sensitivity, specificity and cut-off values have been reported [4–6], but it is very difficult to establish criteria that can be used in routine practice. Several studies have used a single determination of tumour markers in pleural effusions, but establishing cut-off points is problematic – among other reasons, because different types of immunoassay kits obtain different concentrations in the same samples [7].

Equally, the cut-off points may differ according to the aim of the study [8–10].

Another cause of discrepancy between studies is found in the types of benign diseases recorded. It has been reported that tuberculosis, complicated parapneumonic effusions and empyemas may have high concentrations of tumour markers in pleural fluid, related to the inflammation of the mesothelial cells or nearby tissues [11–14].. Adenosine deaminase (ADA), C Reactive Protein (CRP) and granulocyte count are all used in the differential diagnosis of tuberculous effusions, parapneumonic effusions and empyema [15–21].

Paramalignant effusions represent another source of discrepancy. Patients with these effusions have cancer, but no neoplastic cells are present in the pleural mesothelium; however, patients may have high concentrations of tumour markers in serum. So, tumour markers may be found in effusion fluids alongside other macromolecules such as albumin, and they may be present in high concentrations in the pleura. Other benign diseases with high serum concentrations of tumour markers may present similar behaviour [22].

Therefore, in order to obtain high specificity using only values of TM in effusion, high cut-off points have usually been used. However,, our group described a strategy based on two criteria including a low cut-off and the fluid/serum (F/S) ratio [23]. Analysing three types of effusion and using a combination of CEA, CA15-3 and CA19-9, we obtained a sensitivity of 76.2% and a specificity of 97%. Likewise, subclassifying these effusions according to their ADA, CRP and % of polymorphonuclear cells (%PN) value, patients with negative ADA, CRP and %PN obtained a sensitivity of 80% and a specificity of 100% with the F/S ratio >1.2.

The aim of the present study was to compare the diagnostic accuracy of these two strategies: a cut-off point for each tumour marker in fluid effusion to obtain maximum specificity, and the F/S ratio in pleural effusions in order to validate previous results. We also intended to establish whether the classification in groups according ADA, CRP and % of polymorphonuclear cells, might help to improve diagnostic accuracy.

## Methods

From January 2008 to December 2012, fluid and serum samples were collected from consecutive patients of all medical specialties at our center who presented pleural effusions. Diagnostic procedures were performed by assessors who were blind to the study data.

The reference method used was pathological confirmation of cancer in serous effusions or definitive diagnosis assessed during the three months following the determination of TM. Serous effusions were defined as malignant when the presence of neoplastic cells was detected by cytology, biopsy or autopsy. Paramalignant effusions were defined as effusions in which no neoplastic cells were detected by any of the methods described above in patients diagnosed with cancer.

In order to identify benign effusions we determined the following test in fluid and/or serum: protein, albumin, Nt-ProBNP, LDH, microbiological cultures, and if necessary antinuclear antibodies, anti-cyclic citrullinated peptide, rheumatoid factor, thyrotropin, and serological tests for viruses, bacteria and fungi.

Effusion fluid and serum samples were collected and analysed on the same day. CEA, CA15-3, CA 72–4 and CA19-9 were determined using an electrochemiluminescence method on a Cobas 601 analyser (Roche Diagnostics, Barcelona, Spain). The analytical variation expressed as the between-assay coefficient of variation was 5.0, 3.0, 4.2 and 4.5% for CEA, CA15-3, CA72-4 and CA 19–9 at concentrations of 5 μg/L, 32 KU/L, 8.3 KU/L and 29 KU/L respectively. In the first approach, using a single determination in pleural fluid, we established the cut-off for each TM at a specificity of 100% with the ROC curve. For the second approach, simultaneous determinations were performed in fluid and serum; effusions were considered malignant when at least on﻿e of thes﻿e TM CEA, CA15-3, CA72-4 or CA19-9 in fluids were above the URL and the F/S ratio was above 1.2. TM in serum were determined only in patients presenting TM values in pleural fluid above the upper reference limit (URL) in serum (5 μg/L for CEA; 30KU/L for CA15-3; 6.9KU/L for CA72-4, and 37KU/L for CA19-9).

The criteria used to suggest that an effusion might be a false positive (i.e., empyema, complicated parapneumonic or tuberculous) were %PN > 90, CRP > 50 mg/L or ADA >45U/L [20]. The use of the biomarkers ADA, CRP and %PN identified two groups of effusions: group A, effusions with all biomarkers below the cut-off point, and group B, effusions with at least one positive biomarker. ADA (EC3.5.4.4) (ITC Diagnostics, Barcelona, Spain) and CRP (Tina-quant CRP latex, Roche Diagnostics, Barcelona, Spain) were determined in an LX-20 autoanalyser (Beckman Coulter Madrid, Spain). Leukocyte count was performed in a Neubauer chamber and May-Grünwald-Giemsa stain. The analytical variation expressed as the between-assay coefficient of variation was 7.4% for ADA and 2.3% for CRP at concentrations of 10.3 U/L and 76.6 mg/L respectively.

### Statistical analysis

ROC analysis was used to establish cut-off points for each TM at a specificity of 100%.

Sensitivity, specificity, negative predictive values (NPV) and positive predictive values (PPV) were calculated for each TM and for the combination of TM. All statistical analyses were performed using IBM® SPSS® Statistics for Windows v.20 (IBM Corporation, Armonk, New York, USA) and Stata® v.10 (StataCorp LP, College Station, Texas, USA).

## Results

**Fig. 1:**
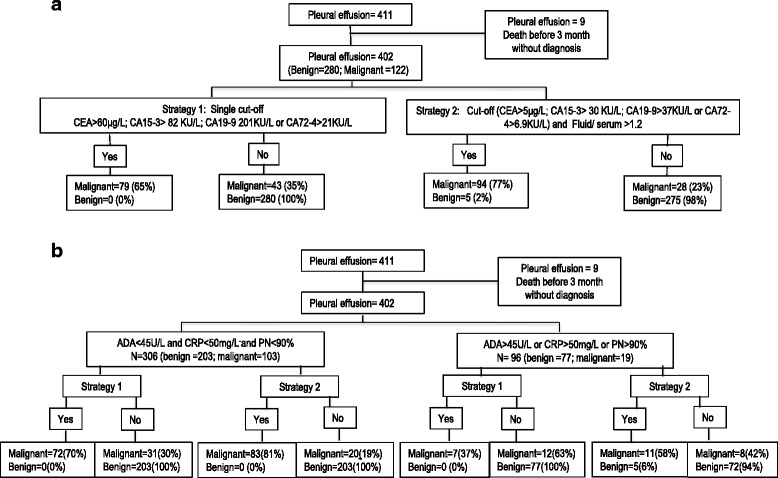
Flow Charts. **a** Flow chart for whole group. **b** Flow chart according ADA, CRP and % polymorphornuclear

A total of 402 consecutive pleural effusions were included, from 148 women and 254 men with ages ranging from 15 to 93 years (mean 72.2; SD 14.2). Out of the effusions assessed, 280 (69.7%) had a benign aetiology and 122 (30.3%) were malignant (Table 1). The effusions were classified in two groups: group A, those with ADA < 45 U/L, CRP < 50 mg/L and %PN < 90%, and group B, those with at least one of the following: ADA >45 U/L, CRP > 50 mg/L and %PN > 90% (Table 1). Figures. 1a and b show the flow charts of participants in this study.

**Table 1:** Etiology of the efusions included in the study

	ALL	ADA<45; CRP<50; %PN<90	ADA>45; CRP>50 or %PN>90
Malignant	122	103	19
Lung cancer	46	40	6
CUP	18	16	2
Mesothelioma	15	12	3
Breast cancer	13	12	1
Lymphoma	9	4	5
Bladder cancer	5	5	0
Ovarian cancer/PSPC^a^﻿	3	3	0
Stomach cancer	3	3	0
Cholangiocarcinoma	2	0	2
Colon cancer	2	2	0
Cervix cancer	1	1	0
Endometrium cancer	1	1	0
Hypernephroma	1	1	0
Melanoma	1	1	0
Multiple myeloma	1	1	0
Pancreas cancer	1	1	0
Cardiogenic	69	56	13
Empyema	11	1	10
Parapneumonic non complicated	26	25	1
complicated	14	2	12
Pneumonitis	10	5	5
Tuberculous	13	0	13
Paramalignant	34	29	5
Viral	10	8	2
Post traumatic	6	6	0
Others: Pulmonary Embolism, Pericarditis, Cirrhotic, nephrotic syndrome, uremia, Rheumatoid arthritis etc.	87	71	16
All	402	306	96

^a^ *PSPC* Papillary serous of peritoneum carcinoma, *CUP* Cancer of unknown primary

The cut-offs with a specificity of 100% obtained with a single determination in pleural effusions were 80 KU/L, 60 μg/L, 209 KU/L and 21 KU/L for CA15-3, CEA, CA19-9 and CA72-4 respectively. For the whole group (Fig. 1a) the sensitivity for all effusions with at least one tumour marker above the cut-off was 63.9%, compared with 51.4% in the group of patients with negative cytology (Tables 2 and 3). The alternative approach (at least one TM above the URL measured in pleural effusion and F/S ratio >1.2) had a sensitivity of 77%, a specificity of 98.2%, an NPV of 90.7% and a PPV of 94.7%; the corresponding results for patients with negative cytology were 71.4, 98.2, 93.2 and 90.9%. When subclassifying according to ADA, CRP, and %PN (Fig. 1b) in group A specificity reached 100% and sensitivities were 69.9% for the whole group and 54.5% in patients with negative cytology using a single cut-off. Using the F/S ratio strategy, specificity reached 100% and sensitivities were 80.6% for the whole group and 72.7% in patients with negative cytology (Tables 2 and 3). In group B, sensitivity fell by more than 6% in both whole group and negative cytology for the two strategies. In addition, using the F/S ratio the specificity fell by more than 6% both in the whole group and in the patients with negative cytology.

**Table 2:** Diagnostic accuracy of tumour markers in effusion

	Single cut-off^a^					Ratio F/S^b^				
	Sensitivity	NPV	Specificity	PPV	Accuracy	Sensitivity	NPV	Specificity	PPV	Accuracy
CEA	36.9	78.1	100.0	100.0	80.6	58.2	84.3	99.0	94.7	85.8
CA15-3	37.7	78.5	100.0	100.0	80.9	43,7	80.2	98.6	92.9	81.6
CA72-4	32.8	77.0	100.0	100.0	79.3	36.1	78.2	100.0	100.0	80.6
CA19-9	22.5	74.5	100.0	100.0	75.8	32.0	77.1	100.0	100.0	79.3
All TM	63.9	86.4	100.0	100.0	89.3	77.0	90.7	98.2	94.7	91.0
Effusions with ADA < 45U/L; CRP < 50 mg/L and %polymorphonuclear cells <90										
CEA	41.7	76.9	100.0	100.0	80.2	63.1	84.0	100.0	100.0	87.2
CA15-3	38.8	76.3	100.0	100.0	79.4	43.7	77.8	100.0	100.0	81.5
CA72-4	34.7	74.3	100.0	100.0	77.4	38.8	76.3	100.0	100.0	79.4
CA19-9	24.8	71.6	100.0	100.0	74.1	34.0	74.8	100.0	100.0	77.7
All TM	69.9	86.8	100.0	100.0	89.9	80.6	91.0	100.0	100.0	93.4
Effusions with ADA > 45U/L; CRP > 50 mg/L and/or %polymorphonuclear cells >90										
CEA	10.5	81.5	100.0	100.0	81.2	31.6	84.9	97.3	75.0	81.2
CA15-3	31.6	85.1	100.0	100.0	86.2	36.8	85.9	94.5	63.6	81.0
CA72-4	18.8	82.2	100.0	100.0	85.6	21.1	83.7	100.0	100.0	84.4
CA19-9	10.5	81.5	100.0	100.0	81.5	21.1	83.7	100.0	100.0	84.4
All TM	36.8	86.5	100.0	100.0	87.5	57.9	90.0	93.5	68.8	85.5

^a^CEA 60 ng/mL; CA15-3 80 KU/L; CA72-4 21 KU/L; CA19-9 201 KU/L

^b^F/S > 1.2 and at least one of these CEA > 5 μg/L, CA15-3 > 30 KU/L, CA72-4 > 6.9 KU/L and CA19-9 > 37 KU/L

**Table 3:** Diagnostic accuracy of tumour markers in patients with negative cytology

	Single cut-off^a^					Ratio F/S^b^				
	Sensitivity	NPV	Specificity	PPV	Accuracy	Sensitivity	NPV	Specificity	PPV	Accuracy
CEA	32.9	85.4	100.0	100.0	86.3	48.6	88.5	99.0	94.4	89.1
CA15-3	24.3	83.9	100.0	100.0	84.7	30.0	84.9	98.6	84.0	84.8
CA72-4	25.0	84.0	100.0	100.0	83.5	28.6	84.8	100.0	100.0	85.1
CA19-9	20.3	82.8	100.0	100.0	84.8	25.7	84.2	100.0	100.0	85.7
All TM	51.4	89.1	100.0	100.0	90.3	71.4	93.2	98.2	90.9	92.8
Effusions with ADA < 45U/L; CRP < 50 mg/L and %polymorphonuclear cells <90										
CEA	40.0	85.8	100.0	100.0	87.1	58.2	89.8	100.0	100.0	91.1
CA15-3	21.8	82.5	100.0	100.0	83.3	27.3	83,5	100.0	100.0	84.5
CA72-4	27.3	82.9	100.0	100.0	83.3	30.9	84.2	100.0	100.0	84.4
CA19-9	24.1	82.4	100.0	100.0	83.9	27.3	83.5	100.0	100.0	85.3
All TM	54.5	89.0	100.0	100.0	90.3	72.7	93.1	100.0	100.0	94.2
Effusions with ADA > 45U/L; CRP > 50 mg/L and/or %polymorphonuclear cells >90										
CEA	10.5	81.5	100.0	100.0	84.3	13.3	85.1	97.4	50.0	83.5
CA15-3	31.6	85.1	100.0	100.0	88.8	40.0	88.9	94.7	60.0	85.7
CA72-4	18.8	82.2	100.0	100.0	83.9	20.0	86.4	100.0	100.0	86.8
CA19-9	10.5	81.5	100.0	100.0	87.2	20.0	86.4	100.0	100.0	86.8
All TM	40.0	86.5	100.0	100.0	90.1	66.7	93.4	93.4	66.7	89.0

^a^CEA 60 μg/L; CA15-3 80 KU/L; CA72-421 KU/L; CA19-9 201 KU/L

^b^F/S > 1.2 and at least one of these CEA > 5 μg/L, CA15-3 > 30 KU/L, CA72-4 > 6.9 KU/L and CA19-9 > 37 KU/L;

Table 4 shows the sensitivity for each strategy according to tumour type and for all tumour markers. The combination of tumour markers allowed detection of more than 80% of the lung, breast, ovarian or bladder cancers and cancers of unknown primary. Other tumours such as lymphoma were not detected.

**Table 4:** Sensitivity of tumour markers according to tumour type

	Single cut-off^a^					Ratio F/S^b^				
Tumour type	CA15-3	CEA	CA19-9	CA72-4	All TM	CA15-3	CEA	CA19-9	CA72-4	All TM
Lung cancer (*n* = 46)	43.5% (20/46)	43.5% (20/46)	28.3% (13/46)	30.4% (14/46)	71.7% (33/46)	36.9% (17/46)	78.2% (36/46)	41.3% (19/46)	39.1% (18/46)	89.1% (41/46)
CUP (*n* = 18)	50.0% (9/18)	72.2% (13/18)	33.3% (6/18)	55,5% (10/18)	94.4% (17/18)	55.5% (10/18)	72.2% (13/18)	50.0% (9/18)	55.5% (10/18)	88.9% (16/18)
Mesothelioma (*n* = 15)	6.7% (1/15)	0.0% (0/15)	0.0% (0/15)	0.0% (0/15)	6.7% (1/15)	46.7% (7/15)	0.0% (0/15)	6.7% (1/15)	0.0% (0/15)	46.7% (7/15)
Breast cancer (*n* = 13)	61.5% (8/13)	7.7% (1/13)	0.0% (0/13)	38.5% (5/13)	92.3% (12/13)	53.8% (7/13)	61.5% (8/13)	0% (0/13)	38.5% (5/13)	84.6% (11/13)
Lymphoma (*n* = 9)	0.0% (0/9)	0.0% (0/9)	0.0% (0/9)	0.0% (0/9)	0.0% (0/9)	0% (0/9)	0% (0/9)	0% (0/9)	0% (0/9)	0% (0/9)
Bladder cancer (*n* = 5)	100% (5/5)	60.0% (3/5)	0.0% (0/5)	40.0% (2/5)	100% (5/5)	60.0% (3/5)	80.0% (4/5)	40.0% (2/5)	40.0% (2/5)	100% (5/5)
Ovarian cancer /PSPC* (*n* = 3)	66.6% (2/3)	33.3% (1/3)	33.3% (1/3)	66.6% (2/3)	100% (3/3)	66.6% (2/3)	33.3% (1/3)	33.3% (1/3)	66.6% (2/3)	100% (3/3)
Stomach cancer (*n* = 3)	33.3% (1/3)	100% (3/3)	100% (3/3)	100% (3/3)	100% (3/3)	33.3% (1/3)	66.6% (2/3)	66.6% (2/3)	100% (3/3)	100% (3/3)
Cholangiocarcinoma (*n* = 2)	0.0% (0/2)	50% (1/2)	50% (1/2)	0.0% (0/2)	50% (1/2)	0% (0/2)	50.0% (1/2)	50.0% (1/2)	0.0% (0/2)	50.0% (1/2)
Colon cancer (*n* = 2)	0.0% (0/2)	50% (1/2)	50.0% (1/2)	50% (1/2)	50% (1/2)	50.0% (1/2)	50.0% (1/2)	0.0% (0/2)	50.0% (1/2)	50.0% (1/2)

^a^Cut-off CEA 60 μg/L; CA15-3 80 KU/L; CA72-421 KU/L; CA19-9 201 KU/L

^b^F/S > 1.2 and at least one of these CEA > 5 μg/L, CA15-3 > 30 KU/L, CA72-4 > 6.9 KU/L and CA19-9 > 37 KU/L

*PSPC* Papillary serous of peritoneum carcinoma, *CUP* Cancer of unknown primar

## Discussion

In our study we evaluated two strategies for assessing tumour markers in pleural effusions. Our results for both strategies were concordant with those of previous publications. With a single determination in pleural fluid, we obtained a joint sensitivity for TM of 63.9% using high cut-off points (60 μg/L for CEA, 80 KU/Lfor CA15-3, 209 KU/Lfor CA19-9 and 21 KU/L for CA72-4). Other studies have shown discriminant values ranging from 40 to 50 μg/L for CEA, 53 to 75 KU/L for CA15-3 and 8.9 to 16 KU/L for CA 72–4 [8, 12, 13, 24], obtaining maximum specificities with sensitivities of between 25 and 45% for each marker individually and between 50 and 70% for the combination. These results are similar to ours. Furthermore, the combined use of URL to detect TM in pleural effusions and the F/S ratio > 1:2 achieved a sensitivity of 77% of and a specificity of 98.2% for the whole group; values similar to those described to those in previous studies conducted by our group with patients presenting pleural, peritoneal and pericardial effusions [23].

The main problem with the use high cut-offs for TM in the differential diagnosis of pleural effusion is the wide range of cut-offs, sensitivities and specificities that we find in the literature, indicating that the discriminant values may depend on the cohort. On this point, considerable differences may be found depending on the type of immunoassay used. Assessing CA15-3, Slev et al. [7]. found concentrations ranging from 8.4 to 16.8 KU/L for Lyphochek low, and from 19.2 to 44.2 KU/L for Lyphochek high. With CYFRA21-1,, Porcel et al. [8] sought high specificity (100%) and obtained a sensitivity of 25% with a cut-off point of 175 μg/L with positive likelihood ratio (LHR+) >9999 and negative likelihood ratio (LHR-) of 0.75, Cynowska et al. [9] sought high sensitivity (90.9%) and obtained a specificity of 7.7% with a cut-off point of 3.3 μg/L with LHR+ of 0.98 and LHR- of 1.18; finally, Korczynski et al. [10], seeking high diagnostic accuracy, obtained a sensitivity of 41.7% and a specificity of 92.1% with a cut-off point of 74.7 μg/L LHR+ of 5.28 and LHR- of 0.63. Likewise, cut-off points ranging between 6.5 and 275 μg/L have been proposed for CEA in order to obtain maximum specificity [20–26].

Comparing the two strategies, the simultaneous determination of TM in serum and effusions and the F/S ratio > 1.2 achieves a 10% higher sensitivity than the single determination, but a lower specificity (98%). In order to improve these data, we assessed the two strategies in group A alone (that is, cases with a low risk of false positives following benign inflammatory processes). In this group, simultaneous determination reached 100% specificity and maintained ranges of sensitivity. Thus, in patients with negative cytology (58% of malignant effusions), sensitivity rose from 54.5 to 72.7%.

Using the F/S ratio and classifying according to ADA, CRP or %PN, we obtained the same specificity in two different studies, and so it seems that this strategy does not vary over time or between series in effusions with negative ADA, CRP and %PN. This strategy uses as a reference the serum concentration measured with the same immunoassay. It avoids some of the problems associated with the single determination of TM, for example the increase in TM concentrations in fluid in patients with high TM concentrations in serum.

Classifying patients on the basis of biomarkers such as ADA, CRP and %PN also allows identification of some benign effusions with high concentrations of TM, thus increasing diagnostic accuracy. The group without suspicion of false positives has higher sensitivity and specificity using either strategy, while in the group of potential false positives sensitivity is much lower with both. In the patients in group B (with at least positive one of the following: ADA, CRP and % of polymorphonuclear cells) included all false positives due to benign release of TM in fluid in both groups, we also found a decrease in sensitivity because this group included fewer malignant effusions and because more than 40% of tumours were non-epithelial, compared with a rate of 18% in group A.

**Fig. 2:**
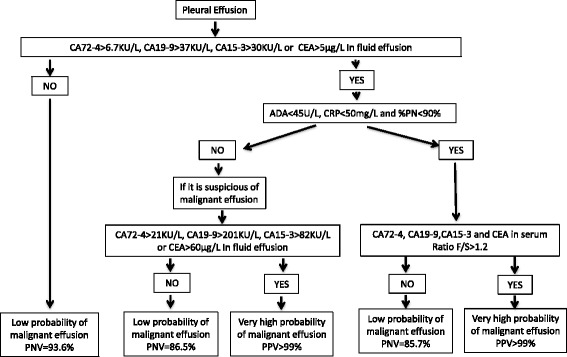
Algorithm for the interpretation of tumour markers in pleural fluid

The data suggest that the best diagnostic accuracy (with very high likelihood ratios) is achieved by using the F/S ratio in group A, that is, patients without suspicion of false positives due to benign diseases. We propose an algorithm for the use of tumour markers in pleural effusions (Fig. 2). The strategy would be to determine tumour markers in fluid effusion; if all are below low cut-off levels (CEA 5 μg/L; CA15-3 30 KU/L; CA19-9 37 KU/L and CA72-4 6.9 KU/L) then the probability of malignant effusion is low. If the fluid effusion concentration is above this cut-off point, ADA, CRP and %PN should be assessed; if at least one is positive, benign disease should be considered. In spite of this, if clinically there is a high suspicion of malignant effusion high cut-offs should be used (60 μg/L, 80 KU/L, 209 KU/L and 21 KU/L for CEA, CA15-3, CA19-9 and CA72-4 respectively). Finally, if ADA, CRP and %PN are negative serum concentrations of TM should be determined and the F/S ratio calculated; if it is above 1.2 there is a very high probability of malignant effusion (LHR+ >999). This is especially important in patients with negative cytology. The use of these criteria can detect three out of four patients with malignant effusion and negative cytology.

Patients with a previous diagnosis of neoplasia and the appearance of pleural effusion with negative cytology and positivity for tumour markers can be taken as presenting disease progression; similarly, the presence of pleural and effusion and positive tumour markers but negative cytology in newly diagnosed localized tumours may indicate pleural metastasis. These and other practical considerations raised by these data should be addressed in a case by case team discussion.

The main limitation of this study is the fact that it was performed at a single centre. Multicentre studies are needed to validate the results and to determine whether they are also applicable to other measurement systems.

## Conclusions

To obtain the maximum diagnostic yield from the measurement of tumour markers in pleural effusions, we support simultaneous determination of markers in fluid and serum with a low cut-off point in patients in whom no increases in TM due to benign disease are suspected (i.e., with ADA, CRP and PN% below the discriminant values). In patients in whom increases in TM due to benign disease are suspected, the strategy of a single determination in fluid may offer better diagnostic accuracy.

## Abbreviations

%PN: Percent of polynuclear cells
ADA: Adenosine deaminase
CA15-3: Cancer antigen 15–3
CA19-9: Cancer antigen 19–9
CA72-4: Cancer antigen 72–4
CEA: Carcinoembryonic antigen
CRP: “c” reactive protein
CUP: Cancer of unknown primary
CYFRA 21–1: Cytokeratin fragment 19
F/S: Fluid/serum ratio
LHR-: Likelihood ratio negative
LHR+: Likelihood ratio positive
PSPC: Papillary serous of peritoneum carcinoma
TM: Tumor markers
URL: Upper reference limit
